# Supplementary material for: Multiblock variable influence on orthogonal projections (MB-VIOP) for enhanced interpretation of total, global, local and unique variations in OnPLS models
Source: BMC Bioinformatics. 2021 Apr 3;22:176. doi: 10.1186/s12859-021-04015-9 (PMC8019512; doi:10.1186/s12859-021-04015-9)
Supplement: Supplementary file 1 — Additional file 1: Supporting information that contains Tables S1–S5 and Figures S1–S5. [file 12859_2021_4015_MOESM1_ESM.docx]

**SUPPORTING INFORMATION** **FOR:**

***Multiblock variable influence on orthogonal projections (MB-VIOP) for enhanced interpretation of total, global, local and unique variations in OnPLS models.***

**Authors:**

Beatriz Galindo-Prieto^a,b,c,*^, Paul Geladi^d^, Johan Trygg^a,e,*^

^a^ Computational Life Science Cluster (CLiC), Department of Chemistry (KBC), Industrial Doctoral School (IDS), Umeå University, Umeå, Sweden
^b^ Department of Engineering Cybernetics (ITK), Norwegian University of Science and Technology (NTNU), Trondheim, Norway

^c^ Helen and Robert Appel Alzheimer’s Disease Research Institute, Feil Family Brain and Mind Research Institute, Weill Cornell Medicine (WCM), Cornell University, New York, USA

^d^ Forest Biomaterials and Technology, Swedish University of Agricultural Sciences (SLU), Umeå, Sweden

^e^ Sartorius Stedim Data Analytics, Umeå, Sweden

* Corresponding authors: beg4004@med.cornell.edu (B. Galindo-Prieto, PhD), [johan.trygg@umu.se](mailto:johan.trygg@umu.se) (J. Trygg, Prof.)

**Contents:**

Tables S1-S5

Figures S1-S5

| **Data** | **Threshold used** | **Number of variables for the total variation** | **Number of variables for the global variation** | **Number of variables for the local variation** | **Number of variables for the unique variation** |  |
| --- | --- | --- | --- | --- | --- | --- |
|  |  |  |  |  |  |  |
| **TRANSCRIPT** | No threshold | 14738 | 14738 | 14738 | 14738 |  |
|  | MB-VIOP ≥ 0.5 | 13127 | 12759 | 10824 | 8368 |  |
|  | MB-VIOP ≥ 1.0 | 4452 | 4451 | 4370 | 3860 |  |
| **PROTEIN** | No threshold | 3132 | 3132 | 3132 | 3132 |  |
|  | MB-VIOP ≥ 0.5 | 2186 | 2175 | 1536 | 0 |  |
|  | MB-VIOP ≥ 1.0 | 683 | 686 | 526 | 0 |  |
| **METABOLITE** | No threshold | 281 | 281 | 281 | 281 |  |
|  | MB-VIOP ≥ 0.5 | 232 | 222 | 168 | 133 |  |
|  | MB-VIOP ≥ 1.0 | 81 | 84 | 68 | 56 |  |

**Table S1:** Number of variables of each omics dataset of the Hybrid Aspen classified by variation type (total, global, local and unique) for the original data (no variable selection threshold applied), for the MB-VIOP selection using threshold ≥ 0.5, and for the MB-VIOP selection using threshold ≥ 1.0.

| **THE 120 MOST IMPORTANT VARIABLES FOR THE TOTAL MODEL (40 VARIABLES PER DATA BLOCK)** | | | | | |
| --- | --- | --- | --- | --- | --- |
| **Transcript**  **variables** | **Total MB-VIOP values (a.u.)** | **Protein variables** | **Total MB-VIOP values (a.u.)** | **Metabolite variables** | **Total MB-VIOP values (a.u.)** |
| PU07944  PU27899  PU22639  PU23318  PU22268  PU28218  PU05769  PU28785  PU25376  PU28089  PU27903  PU28078  PU21598  PU25170  PU27888  PU03044  PU12408  PU28695  PU05015  PU28673  PU22628  PU20341  PU05133  PU22619  PU27826  PU26045  PU25150  PU22573  PU29385  PU27678  PU08985  PU28223  PU10604  PU24177  PU02239  PU23017  PU28528  PU02687  PU29692  PU28093 | 4,48  3,95  3,85  3,84  3,84  3,79  3,75  3,72  3,69  3,68  3,65  3,62  3,56  3,56  3,53  3,52  3,50  3,49  3,47  3,43  3,43  3,38  3,38  3,37  3,35  3,25  3,24  3,23  3,21  3,17  3,17  3,16  3,16  3,16  3,13  3,13  3,12  3,12  3,12  3,12 | 966  1071  3061  795  270  2805  2914  521  2121  29  2481  1125  1644  994  2305  193  527  164  1115  1609  3032  2181  2189  1702  1545  3111  757  206  969  287  934  2086  3118  847  2483  2153  1452  2923  317  1403 | 7,77  6,95  6,07  6,01  5,84  5,68  5,66  5,52  5,46  5,38  5,06  5,06  5,04  4,86  4,67  4,57  4,56  4,49  4,29  4,11  4,04  3,99  3,92  3,91  3,88  3,86  3,74  3,72  3,62  3,57  3,56  3,49  3,38  3,31  3,28  3,26  3,21  3,19  3,19  3,18 | Win022_C04  Win021_C05  Win034_C02  Win025_C01  Win023_C05  Win005_C05  Win034_C04  Win007_C09  Win016_C04  Win018_C10  Win022_C08  Win023_C09  Win013_C03  Win020_C03  Win029_C03  Win031_C01  Win009_C09  Win029_C01  Win013_C11  Win022_C03  Win018_C01  Win017_C02  Win003_C04  Win033_C02  Win015_C08  Win015_C06  Win024_C04  Win007_C13  Win009_C03  Win013_C09  Win019_C03  Win026_C03  Win026_C07  Win001_C02  Win030_C03  Win007_C03  Win002_C04  Win022_C10  Win034_C01  Win034_C03 | 3,38  2,94  2,70  2,63  2,33  2,31  2,26  2,11  2,05  2,01  1,98  1,89  1,83  1,74  1,72  1,70  1,69  1,68  1,67  1,66  1,65  1,62  1,61  1,56  1,54  1,52  1,51  1,48  1,44  1,42  1,39  1,39  1,38  1,38  1,37  1,34  1,32  1,31  1,31  1,30 |

**Table S2:** Identification of the forty most important variables for each block of the Hybrid Aspen data according to their relevance for the total model interpretation (of the original OnPLS model described in Section 2). The total MB-VIOP values are provided in arbitrary units (a.u.).

| **THE 120 MOST IMPORTANT VARIABLES FOR THE GLOBALLY JOINT VARIATION (40 VARIABLES PER BLOCK)** | | | | | |
| --- | --- | --- | --- | --- | --- |
| **Transcript**  **variables** | **Global MB-VIOP values (a.u.)** | **Protein variables** | **Global MB-VIOP values (a.u.)** | **Metabolite variables** | **Global MB-VIOP values (a.u.)** |
| PU07944  PU27899  PU23318  PU22268  PU05769  PU22639  PU25376  PU28089  PU28078  PU25170  PU27888  PU28218  PU28785  PU12408  PU05015  PU27903  PU03044  PU22628  PU05133  PU28673  PU21598  PU27826  PU20341  PU28695  PU26045  PU25150  PU22573  PU10604  PU24177  PU29385  PU22619  PU28223  PU29692  PU02687  PU08985  PU02239  PU27678  PU28528  PU25399  PU23017 | 4,61  4,11  3,99  3,96  3,90  3,90  3,84  3,83  3,77  3,68  3,67  3,64  3,64  3,63  3,62  3,56  3,54  3,53  3,51  3,49  3,48  3,43  3,42  3,39  3,38  3,35  3,33  3,26  3,26  3,26  3,26  3,25  3,24  3,21  3,21  3,20  3,20  3,18  3,18  3,17 | 966  1071  3061  795  270  2914  2805  521  29  2121  1125  2481  1644  994  2305  193  527  164  1609  1115  3032  2181  2189  1702  1545  3111  206  757  934  2086  969  287  3118  847  2153  2483  1452  1403  283  2923 | 7,71  7,03  6,11  6,04  5,90  5,73  5,65  5,58  5,44  5,36  5,12  5,11  5,11  4,88  4,74  4,63  4,54  4,44  4,17  4,13  4,10  4,04  3,96  3,96  3,89  3,88  3,77  3,67  3,61  3,50  3,44  3,41  3,38  3,35  3,30  3,24  3,24  3,22  3,20  3,20 | Win022_C04  Win021_C05  Win034_C02  Win025_C01  Win023_C05  Win005_C05  Win034_C04  Win022_C08  Win018_C10  Win013_C03  Win020_C03  Win009_C09  Win016_C04  Win029_C01  Win013_C11  Win022_C03  Win029_C03  Win018_C01  Win023_C09  Win003_C04  Win017_C02  Win033_C02  Win024_C04  Win031_C01  Win007_C13  Win009_C03  Win026_C03  Win001_C02  Win013_C09  Win026_C07  Win030_C03  Win007_C09  Win019_C03  Win022_C10  Win034_C01  Win002_C04  Win003_C02  Win020_C13  Win015_C06  Win009_C02 | 3,53  2,97  2,81  2,77  2,39  2,39  2,27  2,07  2,03  1,85  1,82  1,78  1,77  1,75  1,73  1,72  1,70  1,70  1,68  1,67  1,66  1,64  1,59  1,56  1,51  1,47  1,45  1,45  1,44  1,43  1,41  1,40  1,39  1,37  1,37  1,35  1,33  1,33  1,29  1,29 |

**Table S3:** Identification of the forty most important variables for each block of the Hybrid Aspen data according to their relevance for the interpretation of the global variation (of the original OnPLS model described in Section 2). The global MB-VIOP values are provided in arbitrary units (a.u.).

| **THE 120 MOST IMPORTANT VARIABLES FOR THE LOCALLY JOINT VARIATION (40 VARIABLES PER BLOCK)** | | | | | |
| --- | --- | --- | --- | --- | --- |
| **Transcript**  **variables** | **Local MB-VIOP values (a.u.)** | **Protein variables** | **Local MB-VIOP values (a.u.)** | **Metabolite variables** | **Local MB-VIOP values (a.u.)** |
| PU06931  PU07326  PU06434  PU03040  PU01604  PU08326  PU30269  PU08307  PU07241  PU07213  PU04361  PU27830  PU31267  PU07802  PU27837  PU28081  PU06797  PU28220  PU07004  PU06101  PU06985  PU07966  PU06614  PU07280  PU30499  PU00630  PU08205  PU31286  PU08286  PU06604  PU22639  PU00660  PU03044  PU05694  PU02114  PU04375  PU06213  PU28218  PU30908  PU08342 | 8,05  6,52  6,21  6,11  5,91  5,76  5,57  5,57  5,55  5,52  5,43  5,41  5,36  5,16  5,01  4,86  4,82  4,69  4,67  4,62  4,61  4,58  4,58  4,53  4,52  4,50  4,46  4,44  4,40  4,39  4,37  4,33  4,33  4,30  4,25  4,25  4,21  4,08  4,07  4,05 | 966  2121  1115  969  287  2805  2368  2364  2969  1991  164  3097  757  2119  121  439  527  795  986  751  2916  3061  718  1119  1960  1829  2124  2483  372  374  435  994  984  1259  1028  2839  625  1297  869  109 | 9,76  8,23  7,97  7,56  7,32  6,84  6,46  6,45  6,43  6,17  5,91  5,90  5,66  5,52  5,33  5,31  5,23  5,18  5,12  4,89  4,88  4,82  4,64  4,53  4,44  4,43  4,35  4,33  4,30  4,27  4,25  4,14  4,09  4,01  4,00  3,97  3,93  3,88  3,88  3,81 | Win031_C01  Win021_C05  Win034_C06  Win034_C04  Win022_C04  Win029_C03  Win018_C01  Win034_C02  Win004_C07  Win022_C05  Win022_C03  Win029_C07  Win013_C11  Win013_C08  Win007_C09  Win008_C08  Win003_C04  Win026_C07  Win032_C01  Win023_C09  Win008_C10  Win020_C14  Win020_C12  Win026_C08  Win014_C02  Win016_C02  Win019_C03  Win015_C02  Win034_C05  Win007_C04  Win020_C18  Win028_C02  Win027_C01  Win024_C07  Win004_C02  Win010_C03  Win020_C11  Win026_C05  Win018_C11  Win006_C01 | 5,39  4,67  4,42  4,23  2,66  2,45  2,35  2,34  2,21  2,18  2,06  2,04  1,93  1,87  1,66  1,62  1,58  1,55  1,50  1,47  1,44  1,44  1,36  1,35  1,33  1,32  1,30  1,29  1,28  1,27  1,25  1,25  1,25  1,24  1,24  1,23  1,22  1,21  1,19  1,18 |

**Table S4:** Identification of the forty most important variables for each block of the Hybrid Aspen data according to their relevance for the interpretation of the local variation (of the original OnPLS model described in Section 2). The local MB-VIOP values are provided in arbitrary units (a.u.).

| **THE 80 MOST IMPORTANT VARIABLES FOR THE UNIQUE VARIATION (40 VARIABLES PER BLOCK)** | | | |
| --- | --- | --- | --- |
| **Transcript**  **variables** | **Unique**  **MB-VIOP values (a.u.)** | **Metabolite variables** | **Unique**  **MB-VIOP values (a.u.)** |
| PU28218  PU27903  PU22619  PU21598  PU28093  PU28695  PU28785  PU22718  PU23246  PU23171  PU26833  PU26977  PU23160  PU05336  PU11583  PU30650  PU23500  PU23219  PU27204  PU27020  PU11487  PU25908  PU27299  PU11448  PU23792  PU22279  PU26882  PU20096  PU08307  PU30561  PU05084  PU23192  PU23215  PU27202  PU11653  PU26956  PU30655  PU07802  PU10587  PU09082 | 6,05  5,92  5,64  5,60  5,43  5,38  5,19  4,74  4,58  4,48  4,39  4,32  4,31  4,25  4,13  4,09  4,04  4,00  3,96  3,93  3,93  3,91  3,91  3,90  3,90  3,90  3,87  3,85  3,84  3,84  3,81  3,80  3,80  3,80  3,76  3,75  3,74  3,74  3,73  3,71 | Win007_C09  Win015_C08  Win016_C04  Win023_C09  Win015_C06  Win014_C02  Win020_C06  Win007_C03  Win022_C02  Win022_C12  Win010_C16  Win018_C10  Win023_C03  Win023_C07  Win020_C12  Win007_C04  Win023_C05  Win021_C05  Win034_C03  Win032_C02  Win011_C02  Win013_C03  Win020_C10  Win007_C10  Win029_C03  Win016_C02  Win016_C05  Win004_C07  Win020_C09  Win005_C05  Win018_C06  Win003_C06  Win007_C08  Win019_C03  Win033_C01  Win009_C10  Win014_C08  Win013_C09  Win021_C03  Win002_C03 | 5,83  4,48  4,25  3,56  3,24  3,16  2,87  2,61  2,19  2,10  2,07  2,02  1,91  1,90  1,89  1,87  1,85  1,85  1,83  1,80  1,76  1,75  1,71  1,70  1,67  1,59  1,56  1,55  1,54  1,49  1,46  1,45  1,34  1,31  1,30  1,29  1,27  1,27  1,25  1,24 |

**Table S5:** Identification of the forty most important variables for each block of the Hybrid Aspen data according to their relevance for the interpretation of the unique model components. The variables of the proteomics data block did not contribute to explain the unique variation of the original OnPLS model, hence, their values are not provided. The unique MB-VIOP values are provided in arbitrary units (a.u.).


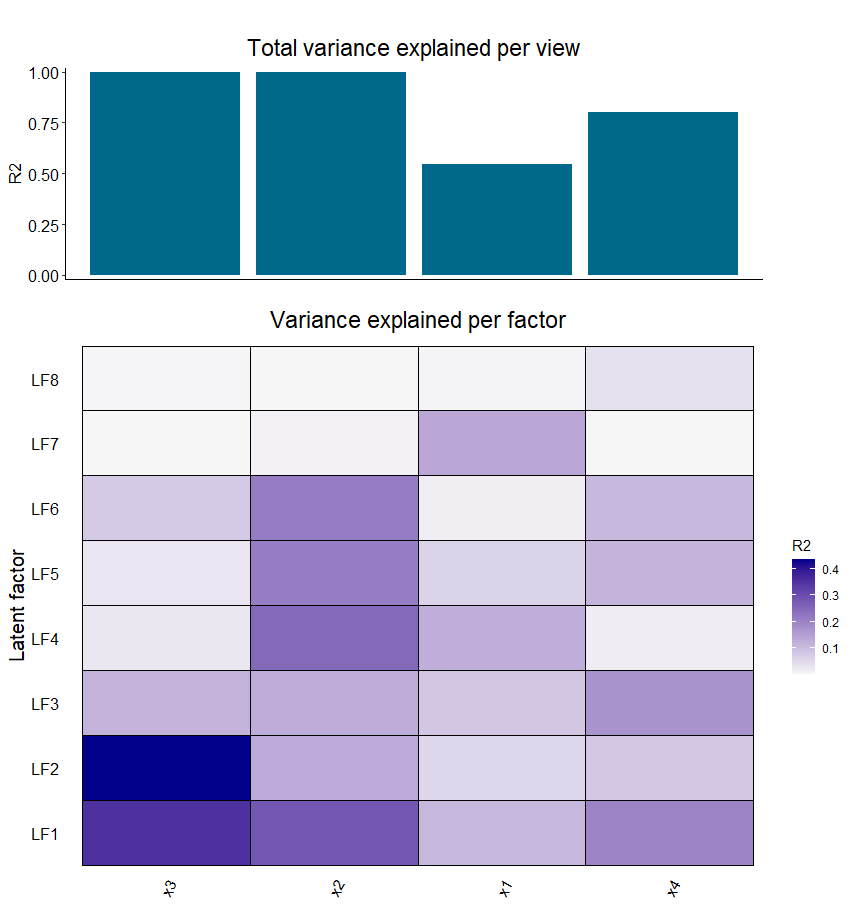


**Figure S1:** Total explained variance for the SD16_235GLU dataset from an 8-component MOFA model.


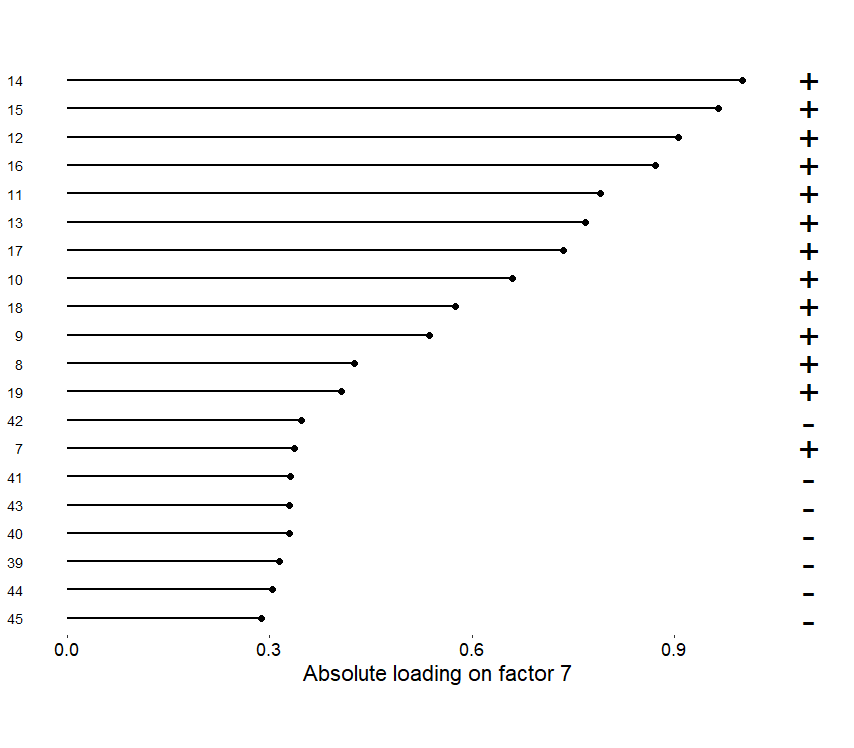


**Figure S2:** Absolute loading plot for the 7^th^ component found by MOFA using the synthetic dataset.


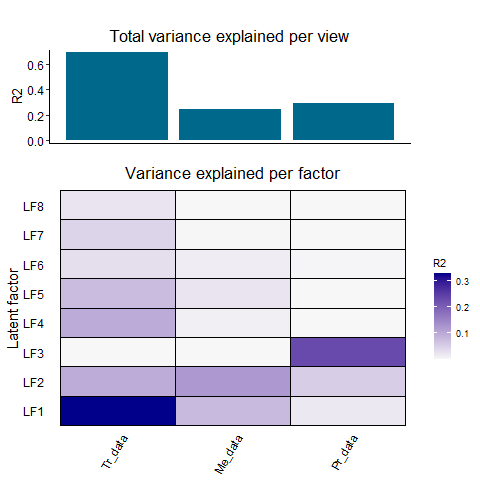


**Figure S3:** Total explained variance for the Hybrid Aspen dataset from an 8-component MOFA model.


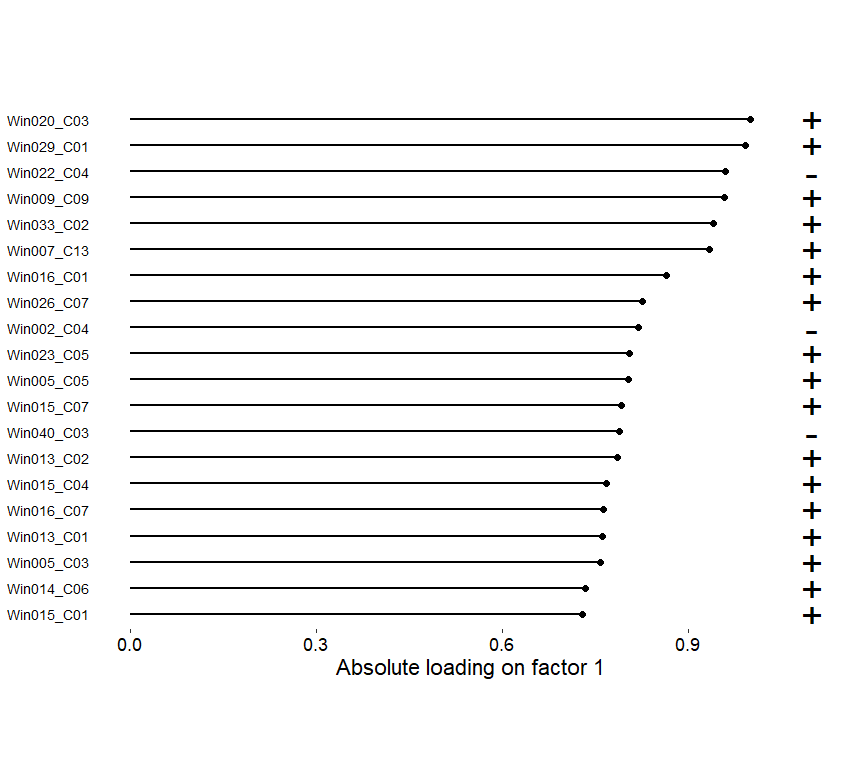


**Figure S4:** Absolute loading plot including metabolite variables for the 1^st^ global component found by MOFA using the Hybrid Aspen dataset.


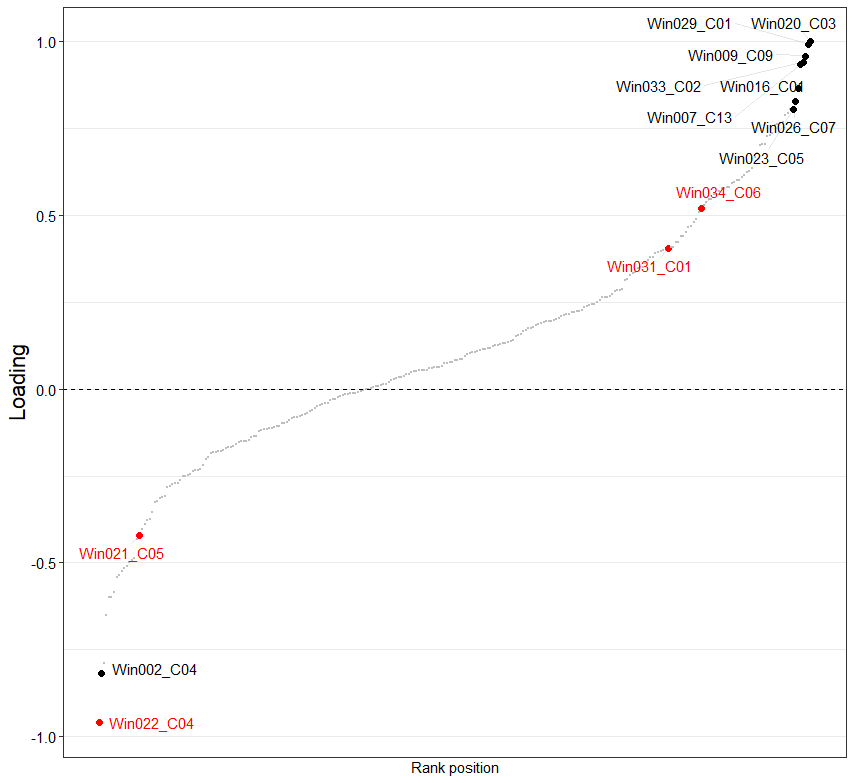


**Figure S5:** Loading vs Rank position plot highlighting some relevant metabolite variables for the 1^st^ global component found by MOFA using the Hybrid Aspen dataset.
